# Health Disparity Measurement Among Asian American, Native Hawaiian, and Pacific Islander Populations Across the United States

**DOI:** 10.1089/heq.2022.0051

**Published:** 2022-07-19

**Authors:** Carolyn Wang Kong, Jennifer Green, Courtnee Hamity, Ana Jackson

**Affiliations:** ^1^ Blue Shield Foundation of California, San Francisco, California, USA.; ^2^ Caduceus Strategies, Sisters, Oregon, USA.

**Keywords:** health disparities, health equity, Asian, Asian American, minority health, measurement

## Abstract

**Objective:**

The aim of this study was to describe current measurement of health disparities for Asian American, Native Hawaiian, and Pacific Islander (AANHPI) populations and subgroups across U.S. states.

**Methods:**

State department of health websites were searched for publicly available online reports and interactive databases denoted as state health or minority health assessments. Sources were examined to determine whether health metrics stratified by any racial/ethnic groups included the AANHPI aggregate population or subgroups. The number and frequency of AANHPI population designations were tabulated, as were the proportion of states that included AANHPIs in stratified metrics in four domains across the life span and the median number of metrics (1) stratified by any racial/ethnic group and (2) including AANHPI populations. A Pearson correlation coefficient assessed the association between the proportion of AANHPIs in state populations and the proportion of state metrics that included AANHPIs in the stratification.

**Results:**

States used 17 AANHPI population descriptors. Of 49 states stratifying health metrics by race/ethnicity, 34 included AANHPI populations and 2 included disaggregated AANHPI subgroups in ≥1 metric. The proportion of states that included AANHPI populations in stratification ranged from 57% for maternal–infant health to 69% for adult health, and by metric groups within domains, the proportion ranged from 14% for maternal mortality to 100% for marital or head of household status. The median number of metrics reported for AANHPI populations was lower than the median number reported for other racial/ethnic groups in adult, maternal–infant, and child and adolescent health domains. The proportion of state metrics that included AANHPIs in racial/ethnic stratification was not correlated with the proportion of AANHPIs in state populations (*r*=0.30).

**Conclusions and Implications for Health Equity:**

AANHPIs were substantially underrepresented in state health equity data, with rare subgroup disaggregation. Reducing disparities and inequities affecting AANHPI health in the United States requires improved and equitable data.

## Introduction

Asians were the fastest growing racial/ethnic population in the United States in 2000–2019, surpassing overall population growth in some states and comprising 20 million people by 2020.^1^ The Native Hawaiian and Pacific Islander populations also grew rapidly during the same time period.^1^ This growth is expected to continue over the next several decades, and identifying and addressing health equity issues for the Asian American, Native Hawaiian, and Pacific Islander (AANHPI) populations will be pivotal to the nation's health.

Health inequities between the aggregate AANHPI population and non-Hispanic Whites in the United States and health disparities across AANHPI subpopulations are well documented at the national level.^2,3^ They include the incidence of stomach, breast, colorectal, liver, and prostate cancers,^4^ proportion of deaths attributable to cancer,^5^ obesity and diabetes prevalence,^6^ tobacco use,^7^ and cardiovascular disease mortality.^8,9^ For example, compared with non-Hispanic Whites in the United States and after controlling for body–mass index, age, and sex, Asians and Pacific Islanders are, respectively, 1.6 and 3.0 times more likely to have diabetes.^10^

Overall rates of cigarette smoking are lower for non-Hispanic Asians than for other population groups,^11^ but range from 7.6% to 20.0% for Asian subgroups.^7^ Factors underlying documented health inequities and disparities are not well understood, but may include access limitations, lack of trust in the health care system, lack of bilingual services, clinician biases, and lack of cultural competence.^3^

Even when inequalities appear to favor AANHPIs, these factors bear consideration. For example, 4.2% of Asian Americans report having had major depression in the past year compared with 9.5% of Whites.^12^ However, Asian Americans are 65% less likely than Whites to be screened for depression by their health care providers.^13^

Health disparities and inequities are primarily documented using survey, registry, and surveillance data reported at the national level, with less data available at the state and local levels. Moreover, national-level reports do not consistently disaggregate the AANHPI population into composite subgroups such as Chinese, Filipino, Japanese, Native Hawaiian, or Samoan.^3^

There is documented value of data disaggregation at state and local levels.^14,15^ Such data are required to accurately assess health disparities and plan interventions. In addition, data disaggregation helps combat the myth of Asian Americans as a single “model minority” uniformly enjoying better health than other populations due to academic and economic success.^14,15^

Assessment is a core governmental public health function.^16^ Accreditation by the national Public Health Accreditation Board, currently held by 40 states,^17^ requires a state health assessment that addresses health disparities and health equity and is available to the general public.^18^ Such assessments form the basis for state health improvement plans and subsequent resource allocation.

However, little is known about how U.S. states assess AANHPI health disparities. The aim of this project was to describe current measurement of health disparities across the life span for the aggregate AANHPI population and AANHPI subgroups across U.S. states.

## Methods

### Design

In August–December 2021, department of health websites in all 50 U.S. states were searched for publicly available online reports and interactive online databases produced by state governments and denoted as state health assessments or minority health data, reports, or assessments. Website searches were conducted with the terms “equity” and “diversity,” supplemented by broad search engine strategies combining individual state names and the terms “health disparities,” “health equity,” and “minority health.”

To assess whether state health assessment reports included any individual-level health measures stratified by at least two racial/ethnic groups, tables of content and figure and table titles were first manually searched for the terms “heath disparity,” “health disparities,” “White,” and “Black.” If no matches were found, a search for the same terms in the entire document was conducted. Documents were excluded if they contained none of these terms.

Sources were excluded if they (1) were older than 10 years, (2) were produced by a nongovernmental entity (e.g., university or nonprofit organization), (3) provided data only for areas smaller than states (e.g., regions or counties), or (4) consisted solely of Behavioral Risk Factor Surveillance System (BRFSS) or vital statistics data. If two or more reports from the same state provided different data on health disparities, the findings were combined.

Included state sources were then examined to determine whether AANHPIs as an aggregate population or specific AANHPI subgroups were included in stratification of individual metrics.

### Health disparity measures and measure uses

AANHPI populations were designated in various ways across and within sources. Each designation appearing in a single source was counted once, even if it was applied to multiple metrics. For example, if a source reported five metrics that included the “American Asian” population and five for the “Asian” population, both designations were counted once.

All individual health disparity metrics stratified for any racial/ethnic population were identified for each state. AANHPIs were identified as included if (1) results for at least one AANHPI population were reported or (2) the report or data repository indicated the intent to provide results for at least one AANHPI population and explicitly stated that results were suppressed due to unreliability or low numbers of events.

Individual metrics fell into four domains: adult health, maternal–infant health, child and adolescent health, and social determinants of health (Table 1; Supplementary Appendix SA1). Within domains, similar metrics were grouped. For instance, within the maternal–infant health category, birth and pregnancy characteristics included cesarean births (primary, in low-risk births, and not otherwise specified), maternal marital status, unintended or intended pregnancy, short interpregnancy interval, and teen births and pregnancies.

**Table 1.** Health Disparity Metric Domains, Groups, and Number of Individual Metrics Identified Across All States

Domain	Metric groups (number of individual metrics within groups)
Adult health	• Chronic disease (108) • Injury and injury-related mortality (67) • Alcohol, substance, and tobacco use (61) • Immunization and infectious disease (60) • Preventive care (52) • Cancer (45) • Mental health (38) • Weight, physical activity, and nutrition (23) • Insurance status and financial barriers to care (20) • Self-reported health (13) • Disability (13) • Mortality and life expectancy (12)
Maternal–infant health	• Prenatal care (21) • Pregnancy or birth characteristics (19) • Maternal tobacco and substance use (19) • Breastfeeding (17) • Infant mortality and morbidity (15) • Low birth weight and preterm births (10) • Maternal weight and weight-related conditions (9) • Postpartum period (4) • Birth and fertility rates (3) • Maternal mortality (3)
Child and adolescent health	• Alcohol, substance use, and tobacco use (92) • Mental health (32) • Weight, physical activity, and nutrition (48) • Risky driving and sexual behaviors (26) • Bullying and violence exposure (25) • Secondhand smoke exposure and smoking risk (19) • Oral health (17) • Exposure to violence (17) • Weight (17) • Mortality (15) • School readiness and performance (10) • Immunization and infectious disease (9) • Access to care (9) • Asthma (9) • Economic and insurance status (8)
Social determinants of health	• Housing burden or security (13) • Education (11) • Economic status (9) • Physical environment (8) • Employment (3) • Marital status (6) • Discrimination (5) • Citizenship status (3) • Transportation (3) • Food access or security (2)

### Analyses

The number and frequency of AANHPI population designations across states were calculated. The number of metrics stratified by race/ethnicity and the number of those that included AANHPI populations were tabulated at the level of individual metrics and aggregated to the group and domain levels. The proportion of states that included AANHPI populations in racial/ethnic stratification was calculated for all metrics and for metric groups within domains.

The median number of reported metrics that were stratified by any racial/ethnic group and the median number of those that include AANHPI populations in stratification were calculated for each domain. Finally, the relationship between the proportion of state populations comprising AANHPIs, based on U.S. Census Bureau data,^19^ and the proportion of state metrics stratified by AANHPI populations was assessed with Pearson's correlation coefficient.

All statistical analyses were performed in Excel (Microsoft).

## Results

Fifty-eight state reports and databases were included: 25 state health assessments or health status reports, 19 health equity or minority health reports, 12 interactive databases, and 2 reports specific to the AANHPI aggregate population (Supplementary Table S1). One source was available for 41 states, 2 were available for 7 states, and 3 were available for a single state. No source was identified for a single state despite multiple searches over several months during the search period.

### AANHPI population groups

Across all included reports and databases, states used 17 different designations for AANHPI populations (Table 2). The most common combination of descriptors was Asian and Asian/Pacific Islander, appearing in sources from 10 states. Population designations typically varied across metrics within sources, and states used an average of two designations. Only two states reported disaggregated data for AANHPI subgroups.

**Table 2.** Asian American, Native Hawaiian, and Pacific Islander Population Designations in Online State Health and Minority Health Reports and Interactive Databases From 49 States

	States using designation, ***N***
Asian/Pacific Islander	23
Asian	17
Asian/Pacific Islander non-Hispanic	9
Asian non-Hispanic	6
Asian Pacific Islander Hawaiian	2
Native Hawaiian	2
Native Hawaiian or other Pacific Islander	2
Other Asian^a^	2
Asian American	1
Asian alone, non-Hispanic	1
Asian/other^b^	1
Pacific Islander	1^c^
Other Pacific Islander	1^d^
Chinese	1^d^
Japanese	1^d^
Filipino	1^d^
Lao/Hmong	1^e^

^a^ Compared with other specific AANHPI groups (e.g., Chinese, Lao/Hmong).

^b^ Including other minority groups.

^c^ California.

^d^ Hawaii.

AANHPI, Asian American, Native Hawaiian, and Pacific Islander.

Metrics that included AANHPI populations in stratification also included at least one other non-White population, such as Blacks/African Americans, Hispanics/Latinos, Native Americans/Alaska Natives, multiracial, or other/unknown.

### AANHPI inclusion in metrics stratified by race/ethnicity

Forty-nine states stratified metrics by racial/ethnic groups, and 36 (73%) of these states included AANHPI populations in at least one stratified metric. A single state included AANHPI populations in all metrics stratified by race/ethnicity.

#### Adult health

Thirty-four (69%) of 49 states that stratified adult health metrics by racial/ethnic groups included AANHPI populations in at least one stratified metric. Across metric groups, 33–58% of states that stratified metrics by any racial/ethnic group included AANHPI populations in one or more metrics (Table 3). States most frequently included AANHPI populations in stratified metrics assessing chronic disease, mental health, and self-reported health and least frequently included them in metrics assessing racial/ethnic disparities in disability and preventive care.

**Table 3.** Proportion of States That Included Asian American, Native Hawaiian, and Pacific Islander Populations in Racial/Ethnic Stratification for Health Disparity Metrics

	AANHPI population included/stratified by any race or ethnicity	%
Adult health		
Chronic disease	28/48	58
Mental health	25/43	58
Self-reported health	11/19	58
Immunization and infectious disease	24/42	57
Injury and injury-related mortality	20/37	54
Cancer	20/39	51
Weight, physical activity, and nutrition	19/41	46
Mortality and life expectancy	13/28	46
Insurance status and financial barriers to care	16/36	44
Alcohol, substance, and tobacco use	16/39	41
Preventive care	13/34	38
Disability	4/12	33
Maternal–infant health		
Birth and fertility rates	5/7	71
Breastfeeding	6/11	55
Prenatal care	18/35	51
Maternal weight and related conditions	3/6	50
Low birth weight and preterm births	19/39	48
Pregnancy or birth characteristics	14/30	47
Infant mortality and morbidity	20/44	45
Postpartum period	3/7	43
Maternal tobacco and substance use	7/21	33
Maternal mortality	1/7	14
Child and adolescent health		
School readiness and performance	11/14	79
Bullying and violence exposure	7/9	78
Oral health	12/17	71
Mental health	12/19	63
Secondhand smoke exposure and smoking risk	3/5	60
Economic and insurance status	7/13	54
Weight, physical activity, and nutrition	12/23	52
Immunization and infectious disease	6/12	50
Risky driving and sexual behaviors	5/11	45
Alcohol, substance, and tobacco use	10/26	38
Asthma	5/14	36
Mortality	3/10	30
Access to preventive care	2/7	29
Social determinants of health		
Marital or head of household status	6/6	100
Transportation	4/5	80
Education	16/22	73
Economic status	19/27	70
Education	16/22	73
Citizenship status	2/3	67
Housing burden or security	7/11	64
Physical environment	4/7	57
Employment	5/10	50
Discrimination	1/2	50
Food access or security	4/9	44

In the 34 states that included AANHPI populations in stratified metrics, a per-state median of 17 adult health metrics was reported for AANHPI populations, compared with a per-state median of 31 metrics for any racial/ethnic group.

#### Maternal–infant health

Of 46 states stratifying maternal–infant health metrics for any racial/ethnic group, 26 (57%) included AANHPI populations in one or more stratified metrics. Across metric groups, 14–71% of states that stratified metrics by any racial/ethnic group included AANHPIs in at least one metric (Table 3). States most frequently included AANHPI populations in stratified metrics assessing birth and fertility rates, breastfeeding, prenatal care, and maternal weight and related conditions and least frequently included them in metrics assessing maternal mortality.

The 26 states that included AANHPI populations in stratified maternal–infant metrics reported a per-state median of 4 metrics for these populations, compared with a per-state median of 5.5 metrics for any racial/ethnic population.

#### Child and adolescent health

Among 41 states stratifying child and adolescent health metrics for any racial/ethnic group, 27 (66%) included AANHPI populations. Across metric groups, 29–79% of states that stratified metrics by any racial/ethnic group included AANHPIs (Table 3). AANHPI populations were most frequently included in racial/ethnic stratification of metrics assessing school readiness and performance and oral health and least frequently included in those assessing access to preventive care and mortality (Table 3).

The 27 states that included AANHPI populations in child and adolescent health metrics reported a per-state median of 5 metrics for AANHPI populations and a per-state median of 6 metrics when including any racial/ethnic group in the stratification.

#### Social determinants of health

Among 34 states stratifying social determinant metrics for racial/ethnic groups, 22 (65%) included AANHPI populations. Across metric groups, the proportion of states that included AANHPI populations in racial/ethnic stratification was 44–100%. AANHPIs were most frequently included in stratification of metrics assessing marital status or head of household and least frequently included in those assessing food access or security.

A median of 4.5 social determinants of health metrics was reported per state for AANHPI populations in the 22 states that included them in the stratification, compared with a per-state median of 5 metrics when including any racial/ethnic stratification.

### AANHPI population size and stratification of metrics

The Pearson correlation coefficient for the relationship between the proportion of state populations comprising AANHPIs and the proportion of metrics stratified by states for AANHPI populations was 0.30, indicating a weak association.

## Discussion

In a survey of publicly available state health equity reports, AANHPI populations were substantially underrepresented. Compared with states stratifying health metrics by any racial/ethnic group, 25% fewer states also included AANHPI populations. Stratification was unrelated to the proportion of state populations comprising AANHPI populations.

Within states reporting AANHPI populations in stratifications, fewer metrics were stratified with AANHPI populations than for other racial/ethnic groups in all but one state. Although substantial health disparities exist across AANHPI subgroups, disaggregated data were available in only two states.

To the best of our knowledge, no reports exist with which we can directly compare our findings, although the need for more high-quality and disaggregated data on AANHPI populations is receiving increasing, although long overdue, attention.^20,21^ For instance, research projects focused on AANHPI participants account for just 0.2% of National Institutes of Health funding.^22^

Several explanations exist for our findings. State reports often draw heavily on federal data sources that fail to measure, reflect, and disaggregate diverse AANHPI experiences.^23^ U.S. Department of Health and Human Services data standards for race/ethnicity collected in federally funded surveys were enhanced under the Affordable Care Act (ACA) to add AANHPI subgroups. Still, data can be rolled up into the more familiar categories that include AANHPIs^20^ and these standards are not required for state reporting.^24^

The size of the aggregate AANHPI population also varies widely across states, which may render calculation of metrics unreliable in states with smaller populations. However, many states did not denote an intent to provide results for one or more AANHPI population groups. Thus, it is unclear whether results were suppressed due to unreliability or low number of events.

Even when population sizes are potentially large enough to reliably stratify results by one or more AANHPI population groups, barriers to representation still exist. Federal surveys providing data used to calculate state metrics, such as the BRFSS, are not available in the languages of many AANHPI subgroups. This is particularly important because linguistic isolation is associated with a greater burden of health disparities and inequities.^25^

Strengths of our project include that it is, to the best of our knowledge, the first to systematically examine the measurement of AANHPI health disparities across states and the included reports resulted from multiple searches through state documents available online for the previous 10 years, reducing the likelihood that state health equity reports were overlooked.

Limitations include the use of data sources available online; states may have additional or updated data sources that were not available online at the time of data collection. In addition, the focused attention that the COVID-19 pandemic required from state health departments may have affected the availability of data.

### Implications for health equity

In health care and health policy, calls are increasing for “measures that matter.”^26^ The lack of health disparity metrics signals the degree to which AANHPI population health is deprioritized relative to the health of other populations. The comparative lack of data at the state level arises from reasons that may include the model minority stereotype.^27^ A conscious or unconscious assumption underlying state analyses may be that no data are needed because no health disparities exist for AANHPIs.

In a vicious cycle, missing data reinforce the stereotype when data users assume these metrics are absent for the same reason.^27^ It is only slightly less deleterious when metrics note that AANHPIs have been omitted due to numbers too small to calculate reliably because a potential interpretation is that any identified disparities would be illusory or affect a population too small to matter.

Importantly, members of AANHPI populations themselves are not immune from this false narrative and may thus avoid engaging in addressing personal or population health concerns. Deprioritizing health disparities for AAHNPI populations also de-emphasizes the importance of cultural competency for clinicians. Finally, state health data are used to identify funding priorities, and the impact of sparse AANHPI data is clear.

The lack of a clear association between the proportion of state populations comprising AANHPIs and the proportion of state metrics stratified by any racial/ethnic group that were also stratified by the AANHPI aggregate population or subgroups has additional implications. Failure to stratify metrics by AANHPI populations cannot be fully explained by populations that are too small to generate statistically reliable comparisons. It raises the question of AANHPI representation in decision-making processes about data incorporated into state health assessments or minority reports.

At a minimum, AANHPI populations should be included in all state data sets to ensure accountability for comparative data that identify disparities in the national AANHPI population. Even when numbers are small, state and local jurisdictions can benefit from counting distinct subgroups whose risk for health disparities may vary.^24^

We are far from the first to emphasize the importance of disaggregating AANHPI data.^25^ Financial constraints, small sample sizes with attendant disclosure risks, and the need to maintain consistent reporting over time are among several factors posing barriers to disaggregation.^25^ However, strategies to address them have been suggested, including federal and state mandates for disaggregation and uniform reporting.^25^

These require significant political will, but the need for data disaggregation extends to all racial/ethnic categories established by the federal Office of Management and Budget: White or Caucasian, Black or African American, Latino or Hispanic, Asian American, Native Hawaiian and Pacific Islander, and American Indian or Alaska Native.^28^

A good starting point for disaggregating AANHPI data is the subgroups established under the ACA: Chinese, Indian, Filipino, Vietnamese, Korean, Japanese, other Asian, Native Hawaiian, Guamanian or Chamorro, Samoan, and other Pacific Islanders.^3^ Best practices for reducing barriers through oversampling and the use of multiple languages, among other strategies, provide guidance for increasing the collection of disaggregated AANHPI data.^25^

## Conclusions

This study demonstrated differences in data available in state health assessments across the United States on health inequities for the aggregate AANHPI population, compared with other population groups, and health disparities across AANHPI subgroups. These differences were not related to the proportion of state populations comprising AANHPIs. Documented health inequities between the aggregate AANHPI population and non-Hispanic Whites and health disparities across AANHPI subgroups exist.

Our findings add important contextual information to persistent calls for more data on the health status of this growing and diverse segment of the population.

## Supplementary Material

Supplemental data

Supplemental data

## Supplementary Material

Supplementary Table S1

Supplementary Appendix SA1

## Abbreviations Used

AANHPI: Asian American, Native Hawaiian, and Pacific Islander
ACA: Affordable Care Act
BRFSS: Behavioral Risk Factor Surveillance System
