# Supplementary material for: Health Disparity Measurement Among Asian American, Native Hawaiian, and Pacific Islander Populations Across the United States
Source: Health Equity. 2022 Jul 19;6(1):533–9. doi: 10.1089/heq.2022.0051 (PMC9518797; doi:10.1089/heq.2022.0051)
Supplement: Supplemental data [file Supp_AppendixSA1.docx]

**Appendix 1.** Construction of health disparity metrics

Individual health disparity metrics were identified in each state, and uses of all identified individual metrics were tabulated across states. For example, adult obesity was identified as an individual metric in the first state where it appeared in a report and recorded on a spreadsheet, with all subsequent uses across states were tabulated. Across states, a total of 1073 individual metrics were identified and collectively used 3025 times.

Consistent with general patterns in state sources, metrics were grouped into four broad categories: maternal-child and women’s health (128 metrics), child and adolescent health (351 metrics), adult health (527 metrics), and social determinants of health (67 metrics).

Within each category, similar metrics were grouped. For example, in addition to adult obesity, related metrics included overweight, obesity or overweight, health body mass index, and healthy body weight. These were grouped into the adult weight category. The number of related metrics within groups varied substantially, from 2 metrics related to sexual or gender minority status to 45 metrics related to cancer incidence and mortality.

The number of metric groups within categories ranged from 11 for social determinants of health to 18 for adult health.
