# Supplementary material for: Health Disparity Measurement Among Asian American, Native Hawaiian, and Pacific Islander Populations Across the United States
Source: Health Equity. 2022 Jul 19;6(1):533–9. doi: 10.1089/heq.2022.0051 (PMC9518797; doi:10.1089/heq.2022.0051)
Supplement: Supplemental data [file Supp_TableS1.docx]

SUPPLEMENTAL TABLE

1. Alabama Community Health Assessment. Montgomery, AL: Alabama Department of Public Health, 2015. Accessed February 14, 2022. <https://www.alabamapublichealth.gov/accreditation/assets/cha2015_final_revaugust_r.pdf>
2. State Health Assessment, 2019. State of Alaska Department of Health and Human Services, Native Tribal Health Consortium. Accessed February 14, 2022. <https://www.healthyalaskans.org/reports/state-health-assessment/>.
3. Alaska Native Health Status Report, 2^nd^ edition. 2017. Alaska Native Tribal Health Consortium Epidemiology Center. <http://anthctoday.org/epicenter/publications/HealthStatusReport/AN_HealthStatusReport_FINAL2017.pdf> Accessed February 14, 2022.
4. Differences in the health status among racial/ethnic groups, Arizona, 2019. Arizona Department of Health Services, 2020. Accessed December 16, 2021. <https://pub.azdhs.gov/health-stats/report/dhsag/dhsag19/index.htm>.
5. Risk Profile of Asians or Pacific lslanders. https://pub.azdhs.gov/health-stats/report/dhsag/dhsag19/pdf/asians.pdf Accessed February 14, 2022.
6. State Health Assessment 2020: Arkansas’s Big Health Problems. Little Rock, AR: Arkansas Department of Health, 2020. Accessed February 14, 2022. https://drive.google.com/file/d/1TtRZQOcf5bUh2-Ed0NQfimkmdYeQBgvX/view
7. Minority Health Publications. Arkansas Department of Health, 2017. Accessed February 14, 2022. https://www.healthy.arkansas.gov/programs-services/topics/minority-health-publications
8. Phillips MM, Quick S, Goodell M. Healthy People 2020 Health Status Report, 2012. Arkansas State Department of Health, Accessed February 14, 2022. https://www.healthy.arkansas.gov/images/uploads/publications/HealthyPeople2020Update.pdf
9. Health Disparities in the Medi-Cal Population: Fact Sheets. California Department of Health Care Services, 2021. Accessed February 14, 2022. https://www.dhcs.ca.gov/dataandstats/reports/Pages/DisparitiesFactSheets.aspx
10. Health Inequities Fact Sheet 2019 Appendix B. Complete Data Tables. Colorado Department of Public Health & Environment, Office of Health Equity, n.d. Accessed January 23, 2022. <https://drive.google.com/file/d/1S0UAQMBZkKiM66JQrvZmByIv7WWtEqIH/view>
11. Health Connecticut 2025: State Health Assessment. Connecticut Department of Public Health, n.d. Accessed January 23, 2022. <https://portal.ct.gov/-/media/Departments-and-Agencies/DPH/dph/state_health_planning/SHA-SHIP/HCT2025/CT_SHA_Report_Final060520.pdf>
12. My Healthy Community: Population Health Dashboard. Wilmington, DE: Delaware Health and Social Services, n.d. Accessed February 14, 2022. https://myhealthycommunity.dhss.delaware.gov/locations/state/community-characteristics
13. 2021 Florida State Health Status. Tallahassee, FL: Florida Department of Health, Accessed December 17, 2021. <http://www.floridahealth.gov/about/state-and-community-health-assessment/ship-process/_documents/2021StateHealthAssessmentDataBook.pdf>
14. State of Georgia State Health Assessment. Georgia Department of Public Health. https://dph.georgia.gov/document/document/georgia-state-health-assessment/download. Accessed January 23, 2022.
15. Hawai’i Health Matters Disparities Dashboard. http://www.hawaiihealthmatters.org/index.php?module=indicators&controller=index&action=dashboard&alias=disparities. Published 2021. Accessed January 23, 2021.
16. Get Healthy Idaho: Leading Health indicators. Idaho Department of Health and Welfare, Division of Public Health. https://www.gethealthy.dhw.idaho.gov/ghi-leadinghealthindicators. Accessed Febuary 4, 2022.
17. Health Disparties Report for Illinois and Illinois Counties: 2011-2015 Data. Illinois Department of Public Health. <https://dph.illinois.gov/content/dam/soi/en/web/idph/files/publications/v5health-disparities-report.pdf>. Accessed January 28, 2021.
18. Indiana State Health Assessment and Improvement Plan: May 2018-December 2021. Indiana State Department of Health. https://www.in.gov/health/files/Indiana_State_Health_Plan_I-SHIP.pdf. Accessed January 28, 2021.
19. Iowa Public Health Tracking Portal. Iowa Department of Public Health. https://tracking.idph.iowa.gov/. Accessed January 28, 2021.
20. Kansas Health Assessment and Improvement Plan. Kansas Department of Health and the Environment. https://www.kdhe.ks.gov/DocumentCenter/View/358/2014-State-Health-Improvement-Plan---Healthy-Kansans-2020-PDF. Published 2014. Accessed December 27, 2021.
21. State Health Assessment Report, 2017 Update. Kentucky Cabinet for Health and Family Services, Kentucky Department for Public Health. https://chfs.ky.gov/agencies/dph/Documents/StateHealthAssessment.pdf. Accessed February 1, 2022.
22. MInority Health Indicators. Louisiana Department of Health. https://ldh.la.gov/index.cfm/page/672. Accessed February 4, 2022.
23. Maine Interative Health Data, Maine Shared Community Health Needs Assessment. Maine Department of Health and Human Services. https://www.maine.gov/dhhs/mecdc/phdata/MaineCHNA/maine-interactive-health-data.shtml. Accessed February 14, 2022.
24. Maryland State Health Improvement Process. Maryland Department of Health. https://health.maryland.gov/pophealth/Pages/SHIP-Lite-Home.aspx. Accessed February 14, 2022.
25. 2017 Massachusetts State Health Assessment. Massachusetts Department of Health. https://www.mass.gov/files/documents/2017/11/03/2017%20MA%20SHA%20final%20compressed.pdf. Accessed December 27, 2021.
26. Health Equities Dashboard. Massachusetts Department of Public Health, Office of Health Equity. https://app.powerbigov.us/view?r=eyJrIjoiM2Y0YmEwZmMtMzAzNi00YTc1LTg4YjQtZmRmODJmMjllNDQzIiwidCI6IjNlODYxZDE2LTQ4YjctNGEwZS05ODA2LThjMDRkODFiN2IyYSJ9&pageName=ReportSection95d1c2ee2de9266c7922 Accessed February 2, 2022.
27. 2020 Health Equity Report: Moving Health Equity Forward. Michigan Department of Health and Human Services. https://www.michigan.gov/documents/mdhhs/2020_PA653-Health_Equity_Report_Full_731810_7.pdf.
28. 2. Summary Data Brief of the Changes in Health Disparties Between 2010-2019. Michigan Department of Public Health. https://www.michigan.gov/documents/mdhhs/PA653_Databrief_Layout_731811_7.pdf.
29. 2017 Minnesota Statewide Health Assessment. Minnesota Department of Health. https://www.health.state.mn.us/communities/practice/healthymnpartnership/docs/2017MNStatewideHealthAssessment.pdf. Published 2019. Accessed December 27, 2021.
30. Minnesota Public Health Data Access. MInnesota Department of Health. https://data.web.health.state.mn.us/web/mndata. Accessed December 17, 2021.
31. State of the State: Annual Mississippi Health Disparities and Inequalities Report. MIssissippi State Department of Health. http://www.msdh.state.ms.us/msdhsite/index.cfm/44,8072,236,63,pdf/HealthDisparities2019.pdf. Published 2018. Accessed December 27, 2021.
32. Missouri Information for Community Assessment: Minority Health. Missouri Department of Health and Senior Services. https://healthapps.dhss.mo.gov/MoPhims/ProfileHome. Accessed December 27, 2021.
33. Montana State Health Assessment 2017: A Report on the Health of Montanans. Montana Department of Health and Human Services. https://dphhs.mt.gov/assets/publichealth/ahealthiermontana/2017SHAFinal.pdf. Accessed December 27, 2021.
34. Nebraska Health Disparities Report: Health Equity for All Nebraskans. Department of Health and Human Services. https://dhhs.ne.gov/Reports/Health%20Disparities%20Report%202020.pdf. Published 2020. Accessed December 27, 2021.
35. Nebraska Minorities Disparities Facts Chart Book. Department of Health & Human Services. https://dhhs.ne.gov/Reports/Nebraska%20Disparities%20Chartbook%202021.pdf. Published 2021. Accessed December 27, 2021.
36. Minority Health Report. Office of Analytics, Department of Health and Human Services, Nevada. http://dhhs.nv.gov/uploadedFiles/dhhsnvgov/content/Programs/Office_of_Analytics/Images/Minority%20Health%20Report%202019%20v1.0.pdf. Published 2019. Accessed December 27, 2021.
37. Health Equity Report Card. New Hampshire Health and Human Services. . Accessed February 14, 2022.
38. Health Disparity Priority Areas Indicator Report Index. New Jersey Department of Health. https://www-doh.state.nj.us/doh-shad/indicator/CatDisparity.html. Accessed December 28, 2021.
39. Health Equity in New Mexico 13th Edition. New Mexico Department of Health. https://www.nmhealth.org/publication/view/report/2045/. Published 2019. Accessed December 28, 2021.
40. New York State Health Equity Report: County Edition, April 2019. New York State Department of Health. https://www.health.ny.gov/community/minority/docs/health_equity_report_2019.pdf. Accessed December 28, 2021.
41. Racial and Ethnic Health Disparities In North Carolina: North Carolina Health Equity Report 2018. NC Department of Health and Human Services, Office of Minority Health and Health Disparities. https://schs.dph.ncdhhs.gov/SCHS/pdf/MinorityHealthReport_Web_2018.pdf. Accessed December 28, 2021.
42. North Dakota 2017 Health Disparities Report. North Dakota Department of Health. https://www.health.nd.gov/sites/www/files/documents/Files/HSC/HEO/Health_Equity_Report.pdf. Accessed December 28, 2021.
43. 2019 Online State Health Assessment. Ohio Department of Health. https://odh.ohio.gov/wps/portal/gov/odh/explore-data-and-stats/interactive-applications/2019-Online-State-Health-Assessment. Accessed December 28, 2021.
44. 2019 Oklahoma Minority Health at a Glance. Oklahoma Department of Health. https://oklahoma.gov/content/dam/ok/en/health/health2/documents/2019-oklahoma-minority-health-at-a-glance.pdf. Published 2019. Accessed December 28, 2021.
45. Demographics: State of the State’s Health Report. Oklahoma State Department of Health. https://stateofstateshealth.ok.gov/Data/Demographic. Published 2021. Accessed December 28, 2021.
46. Oregon’s State Health Assessment. Oregon Health Authority, Public Health Division. https://www.oregon.gov/oha/PH/ABOUT/Documents/sha/state-health-assessment-full-report.pdf. Published 2018. Accessed December 28, 2021.
47. The State of Our Health: A Statewide Health Assessment of Pennsylvania. https://www.health.pa.gov/topics/Documents/Health%20Planning/SHA%20Complete%20Report_2021.pdf. Accessed December 27, 2021.
48. Rhode Island State Health Improvement Plan, Component A: Health Assessment Report. State of Rhode Island Department of Health. https://health.ri.gov/publications/reports/2017StateInnovationModelHealthAssessment.pdf. Published 2017. Accessed December 27, 2021.
49. South Carolina State Health Assessment. South Caroline Department of Health and Environmental Control. https://livehealthy.sc.gov/sites/default/files/Documents/SHA%20chapters/sc_sha_full_report_nov.18.pdf. Published 2018. Accessed December 28, 2021.
50. Tennesse’s Vital Signs. Tennesee Department of Health. https://www.tn.gov/health/health-program-areas/tennessee-vital-signs/redirect-tennessee-vital-signs/redirect-dashboard/dashboard.html. Accessed December 28, 2021
51. The Health Status of Texas 2014. Department of State Health Services. https://www.dshs.texas.gov/chs/HealthStatusTexas2014.pdf. Accessed December 28, 2021.
52. Utah State Health Assessment 2019. Utah Department of Health Office of Public Health Assessment. https://ibis.health.utah.gov/ibisph-view/pdf/opha/publication/SHAReport2019.pdf. Accessed December 28, 2021.
53. Vermont State Health Assessment. Vermont Department of Health. https://www.healthvermont.gov/sites/default/files/documents/pdf/VT%20State%20Health%20Assessment%202018%20Full%20Report.pdf. Published 2018. Accessed December 30, 2021.
54. Virginia Health Equity Report. Commonwealth of Virginia Department of Health. https://www.vdh.virginia.gov/content/uploads/sites/76/2016/06/Health-Equity-Report-2012.pdf. Published 2012. Accessed December 30, 2021.
55. Washington State Health Assessment. Washington State Department of Health. https://www.doh.wa.gov/Portals/1/Documents/1000/2018SHA_FullReport.pdf. Published 2018. Accessed December 28, 2021.
56. 2012 West Virginia State Health Profile. West Virginia Department of Health and Human Services. https://dhhr.wv.gov/publichealthquality/statepublichealthassessment/Documents/2012%20State%20Health%20Profile%20Final%20May%202013.pdf. Accessed December 30, 2021.
57. Healthiest Wisconsin 2020 baseline and health disparities report (Apr. 18, 2014). Wisconsin Department of Health Services. https://www.wistatedocuments.org/digital/collection/p267601coll4/id/10140/. Accessed December 30, 2021.
58. Racial and Ethnic Disparities in Wyoming: 2012 Report: Wyoming Department of Health Public Health Division. <https://health.wyo.gov/wp-content/uploads/2016/04/43-13358_RacialandEthnicDisparities.pdf>. Accessed December 17, 2021.
